# Case report: Continuous infusions of ceftazidime-avibactam and aztreonam in combination through elastomeric infusors for 12 weeks for the treatment of bone and joint infections due to metallo-β-lactamase producing *Enterobacterales*

**DOI:** 10.3389/fmed.2023.1224922

**Published:** 2023-08-03

**Authors:** Yanis Merad, Anne Conrad, Sophie Brosset, Axel Schmidt, Camille Hanriat, Sebastien Lustig, Frederic Laurent, Camille Kolenda, Tiphaine Roussel-Gaillard, Cecile Batailler, Tristan Ferry, Lyon BJI Study group

**Affiliations:** ^1^Infectious Disease Department, Hôpital de la Croix Rousse, Hospices Civils de Lyon, Lyon, France; ^2^Université Claude Bernard Lyon 1, Lyon, France; ^3^International Center for Research in Infectiology, INSERM U1111, Université Claude Bernard Lyon 1, CNRS, UMR5308, Ecole Normale Supérieure de Lyon, Univ Lyon, Lyon, France; ^4^Centre Interrégional de Référence pour la Prise en Charge des Infections Ostéo-Articulaires Complexes (CRIOAc Lyon), Hospices Civils de Lyon, Lyon, France; ^5^Plastic and Reconstructive Surgery Department, Hôpital de la Croix Rousse, Hospices Civils de Lyon, Lyon, France; ^6^Orthopaedic and Traumatology Surgery Departement, Hôpital de la Croix Rousse, Hospices Civils de Lyon, Lyon, France; ^7^Institut des Agents Infectieux, Laboratoire de Bactériologie, Centre National de Référence des Staphylocoques, Hôpital de la Croix-Rousse, Hospices Civils de Lyon, Lyon, France

**Keywords:** antibiotic resistance, ceftazidime-avibactam, aztreonam, *Enterobacterales*, bone and joint infections, metallo-beta-lactamase, elastomeric infusor

## Abstract

Among carbapenem-resistant *Enterobacterales*, metallo-beta-lactamase producing strains represent a growing therapeutic challenge. While the association of aztreonam and ceftazidime-avibactam has been investigated in recent years for the treatment of infections involving these strains, little to no clinical data support the use of this association for the treatment of bone and joint infections. We report two cases of complex bone and joint infections involving metallo-beta-lactamase-producing *Enterobacterales*, successfully treated at our referral center with aztreonam and ceftazidime-avibactam for 12 weeks in continuous infusions through elastomeric infusors.

## Introduction

Antimicrobial resistance is a major and growing cause of mortality in the 21st century. While it was responsible for 0.7 million deaths in 2014, this estimate is expected to grow up to 10 million deaths annually by 2050 (1). Extended-spectrum beta-lactamase (ESBL)-producing and carbapenem-resistant *Enterobacterales* are recognized as priority targets for the development of new antibiotic strategies. The development of new antibiotic molecules has been increasing in the last 10 years, after being at its lowest around 2010, but the introduction of new antibiotics is bound to select new resistance mechanisms among bacteria (2).

The main mechanism associated with β-lactam resistance in *Enterobacterales* is the production of β-lactamases, which are enzymes capable of hydrolyzing the β-lactam cycle. They are traditionally divided into four groups according to Ambler's classification. Carbapenem-resistant *Enterobacterales* strains most frequently produce one or several β-lactamases from either Ambler class A, B, and/or D (3). Class B β-lactamases are metallo-β-lactamases (MBL), which are all carbapenemases (e.g., VIM, IMP, and NDM). Of note, as these strains disseminate and are more and more responsible for severe infections, the use of novel antimicrobial strategies is required (4), especially to treat MBL-producing *Enterobacterales*.

In recent years, several innovative strategies have emerged to treat carbapenem-resistant *Enterobacterales* (5). On the one hand, antimicrobial combinations associating “old” β-lactams with “new” β-lactamase-inhibitors, including avibactam (AVI), have been developed—as traditional ones, such as clavulanic acid and tazobactam, cannot inhibit the hydrolytic properties of carbapenemases. These new β-lactamase-inhibitors have been associated with broad-spectrum cephalosporins or carbapenems with proven *in vitro* and *in vivo* restored activity against carbapenem-resistant *Enterobacterales* strains. However, these combinations are not active against MBL. On the other hand, new β-lactam molecules, such as cefiderocol, with intrinsic activity against carbapenemase-producing strains have been developed (6). However, these new strategies have inconsistent efficacy for MBL-producing *Enterobacterales* eradication (7, 8). Such infections remain particularly difficult to treat, with no active β-lactam/β-lactamase-inhibitor combination approved for clinical use to date and an inconsistent susceptibility to siderophore cephalosporins such as cefiderocol.

Aztreonam is a β-lactam of the monobactam family. Its antimicrobial spectrum includes a wide range of gram-negative bacteria. Usually, this antibiotic is mainly used for the treatment of gram-negative bacteria infections in patients allergic to penicillin or cephalosporins as allergic cross-reactivity with aztreonam almost never occurs (9). Interestingly, aztreonam is not hydrolyzed by MBL, but MBL-producing strains are usually resistant to aztreonam due to the frequent co-production of other β-lactamases (e.g., ESBLs, serine-carbapenemases, or OXA-like carbapenemases), which hydrolyze aztreonam (10).

Hereby, the combination of aztreonam (ATM) with a broad-spectrum β-lactamase-inhibitor (e.g., AVI, only available for clinical use through the ceftazidime-avibactam combination) has recently emerged as a last resort treatment option for MBL-producing *Enterobacterales* and has been investigated in recent *in vitro* studies. *In vitro* susceptibility rate of MBL-producing Enterobacterales strains to ATM and CAZ-AVI in association ranges from 80 to 100% of strains in most reported series (10–12). Nevertheless, there is still little clinical evidence supporting the use of the association of ATM with AVI (or CAZ-AVI) in a clinical setting, notably for infections with issues concerning the poor diffusion of antibiotics and the length of treatment such as bone and joint infections (BJI) (12–14). Moreover, ATM and CAZ-AVI are antibiotics that are frequently prescribed and are administered at the dose of 2 g and 2 g/0.5 g, respectively, every 8 h as discontinuous therapy, but as they are time-dependent antibiotics (as other beta-lactam antibiotics), it would be relevant to perform continuous infusions of these antibiotics to optimize the time above the minimum inhibitory concentration (MIC) of the targeted pathogen. Regarding the stability of these drugs, each of them was considered to be stable in elastomeric infusors during 12 h (15). Elastomeric infusors are useful to treat patients in the outpatient setting, and their use should be considered in the setting of complex infections such as BJI.

The aim of this case series is to illustrate the combined use of ATM and CAZ-AVI in two patients with BJI involving MBL-producing *Enterobacterales* who were treated recently at our referral center.

## Methods

The CRIOAc Lyon (https://www.crioac-lyon.fr/en) is a French referral center for the management of complex BJI. Since the setup of the ongoing prospective cohort study called the Lyon BJI cohort study in 2017 (NCT02817711) that includes all patients managed in our center, two patients infected with MBL-producing *Enterobacterales* strains were treated with ATM and CAZ-AVI. They were identified through a keyword screening of the database that included more than 5,000 patients.

This study was reviewed and approved by Hospices Civils de Lyon Ethics Committee under the MR004 regulation (N°22-5034). All patients were informed of this study, and their consent for the use of their medical data was sought in an opt-out design.

Susceptibility testing was performed using Vitek 2 (BioMérieux, Marcy l'Etoile, France), and the minimal inhibitor concentrations for some antibiotics were determined using UMIC tests (Biocentric, Bandol, France) or E-test strips (Biomérieux) according to CASFM/EUCAST guidelines. The MIC of the combination of ATM/CAZ-AVI was measured using a CAZ-AVI strip placed onto the agar for 10 min after inoculation and then replaced by an ATM strip (strip superposition method) (16). A representative figure showing the results of MIC testing is shown in Supplementary Figure 1. Carbapenemase production was assessed using the multiplex PCR (Xpert Carba-R^®^, Cepheid, Sunnyvale, USA) or immunochromatographic test (Resist, Coris BioConcept, Gembloux, Belgium).

## Case 1

**Figure 1:**
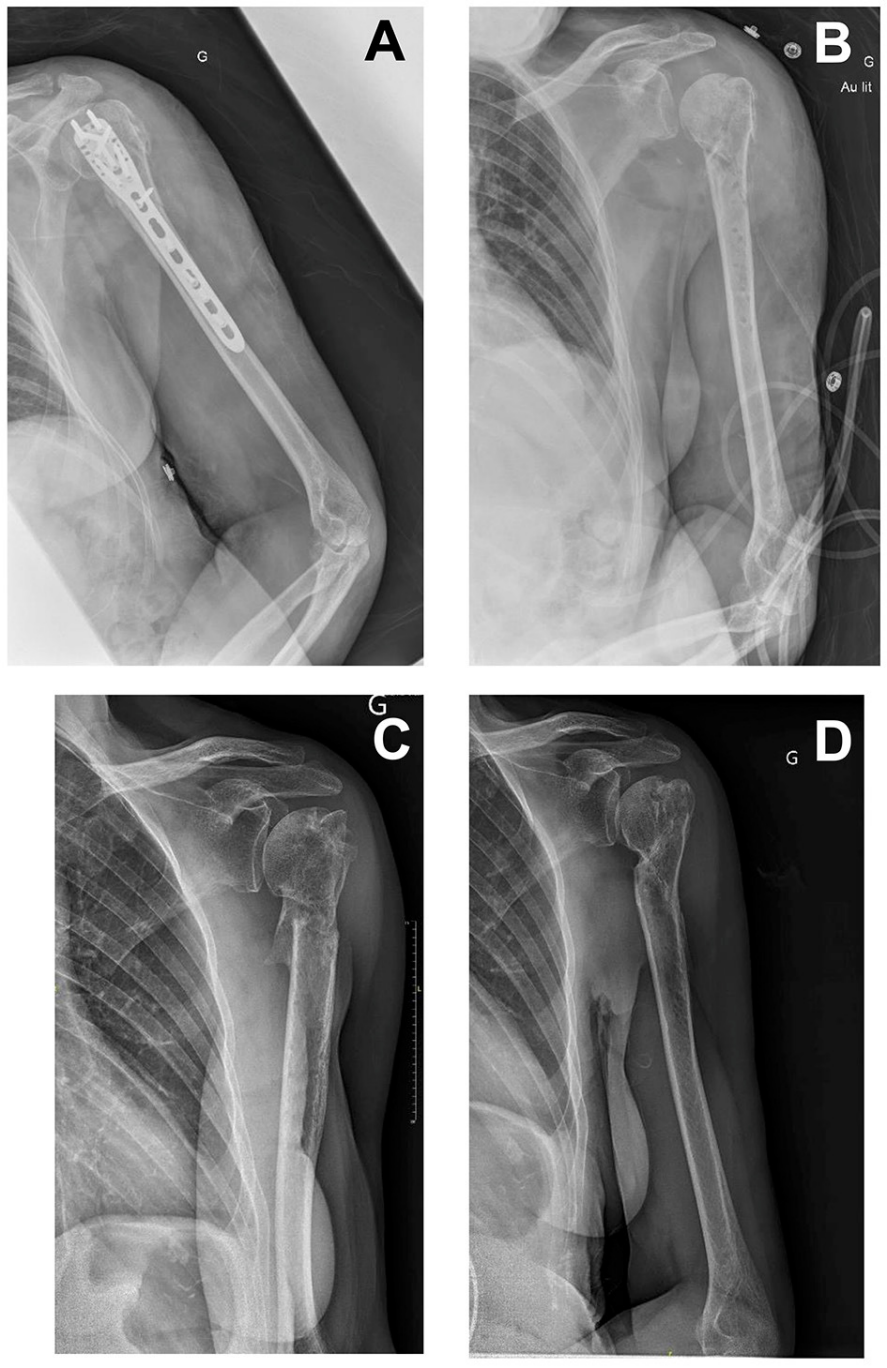
**(A)** An X-ray image of 2 days before debridement and implant removal surgery. **(B)** An X-ray image immediately after debridement and implant removal surgery. **(C)** An X-ray image of 6 months after surgery. **(D)** An X-ray image of 18 months after surgery.

A 70-year-old woman who had a history of breast cancer was in complete remission for 8 years and had essential hypertension. She was a victim of a traffic accident during a leisure vacation in Uzbekistan and suffered multiple traumas, including a left humeral diaphyseal fracture. She was first cared for in Turkey, where she underwent humeral plate osteosynthesis. She required a stay of several days in intensive care, during which she developed ventilation-acquired pneumonia, seemingly with a carbapenem-resistant strain (although we were not able to retrospectively collect the microbiological results obtained in the Turkish lab), requiring treatment with colistin and tigecycline. Upon sanitary repatriation to our referral center 32 days later, her surgical wound was found to be inflammatory, with a purulent discharge highly evocative of surgical site infection. Therefore, she underwent surgical site debridement and implant removal 35 days after the first surgery (see post-operative X-ray in Figure 1). Pre-operative findings were an abscess around the osteosynthesis and showed no sign of fracture healing. Multiple microbiological samples were collected during surgery. Immediately after surgery, the patient was started on empiric antibiotic therapy associated with vancomycin, cefepime, and metronidazole, and quickly switched on day 1 to meropenem, colistin, and daptomycin.

An NDM-producing *Klebsiella pneumoniae* strain was cultured from all pre-operative samples. Antibiotic susceptibility testing notably found it to be resistant to meropenem (MIC > 32 mg/L), colistin (MIC:64 mg/L), levofloxacin, fosfomycin, and co-trimoxazole but susceptible to tigecycline (MIC: 0.25 mg/L). No other bacteria were found in the culture. Upon these findings, meropenem and colistin were stopped on day 3, and she was started on tigecycline. Further tests showed that the MIC of ATM combined with CAZ-AVI was 0.19 mg/L, while ATM and CAZ-AVI individual MICs were ≥ 256 mg/L. Therefore, tigecycline was discontinued, and she was started on a triple antibiotic therapy including ATM (continuous infusion of 3 g in 150 ml of NaCl 0.09% per 12 h, twice a day), CAZ-AVI (continuous infusion of 3 g/0.75 g in 150 ml of NaCl 0.09% per 12 h, twice a day), and delivered as a continuous infusion using elastomeric infusor and fosfomycin (3 g/8 h, discontinuous infusion).

The postoperative outcome was favorable: there was no clinical relapse, and inflammatory biomarkers decreased over the weeks following surgery. As a result, fosfomycin was discontinued 4 weeks after surgery, allowing the patient to be discharged from the hospital. ATM and CAZ-AVI were administered for a total of 12 weeks after surgery. There was no adverse effect of this antibiotherapy.

Follow-ups at 3, 6, 9, 18, and 24 months showed favorable outcomes. The humeral fracture was healed at 6 months. There was a slight limitation of abduction angle, not associated with any significant loss of functional ability.

## Case 2

**Figure 2:**
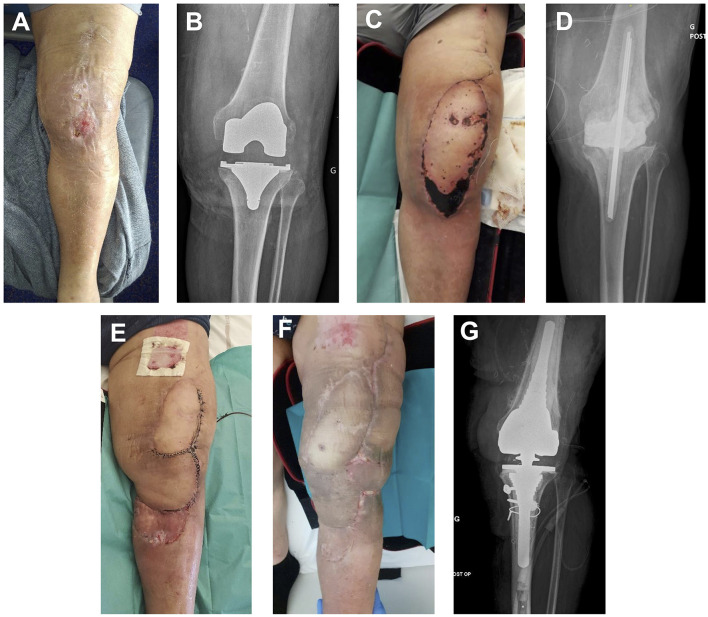
**(A)** Initial presentation after 10 days of ceftobiprole for left knee cellulitis. **(B)** X-ray at initial presentation. **(C)** Thigh flap partial necrosis 10 days after implant removal surgery. **(D)** X-ray after implant removal surgery. **(E)** Second surgery: spacer replacement, rotation flap, and STSG. **(F)** Clinical presentation 2 months after last surgery (total knee arthroplasty reimplanted). **(G)** X-ray at 12 months follow-up after total knee arthroplasty reimplantation.

A 71-year-old man had a history of obesity (100 kg), with essential hypertension, hypercholesterolemia, right knee total arthroplasty, and bilateral total hip arthroplasty. He underwent total left knee arthroplasty, with early post-operative infection caused by methicillin-susceptible *Staphylococcus aureus* (MSSA), methicillin-resistant *Staphylococcus epidermidis* (MRSE), *Streptococcus agalactiae*, and *Finegoldia magna*. Despite two iterative debridement surgeries and adequate antibiotic therapy for 6 months, the outcome was not favorable, and he showed clinical symptoms of relapsing left knee infection 1 month after antibiotic discontinuation. He had left knee dermohypodermitis, and there was a purulent discharge through a fistula in direct continuity with the prosthetic articulation. He received 10 days of ceftobiprole, which successfully cured dermohypodermitis. He then underwent the first surgery of a two-stage left knee arthroplasty replacement strategy (see pre-operative photography and post-operative X-ray in Figure 2). The implant was removed, soft tissues were debrided, and a non-articulated spacer was molded with gentamicin- and vancomycin-loaded polymethylmethacrylate cement (COPAL^®^ G+V). An anterolateral thigh flap was performed. Empiric post-operative antibiotherapy was daptomycin, cefepime, and metronidazole. Definitive antibiotic therapy with co-trimoxazole and rifampicin was started upon finding only MSSA in all pre-operative sample cultures at day 15.

Unfortunately, he developed necrosis on the distal portion of his flap, prompting him to perform a new surgery 1 month after the previous one. Soft tissues were debrided, and an intra-articular collection was evacuated; multiple samples were collected. The spacer was removed, and a new one was implanted (COPAL^®^ G+V with manual addition at the time of spacer preparation of 2 g of fosfomycin). A new flap and a split-thickness skin graft were performed. Post-operative empiric antibiotherapy was piperacillin-tazobactam and daptomycin. An NDM and OXA-48-producing *Enterobacter cloacae* strain was cultured in all samples. It was resistant to meropenem, levofloxacin, and co-trimoxazole. It had intermediate susceptibility to tigecycline (MIC: 1.5 mg/L). It was susceptible to colistin (MIC: 1 mg/L), fosfomycin (MIC:12 mg/L), and amikacin. Moreover, it was resistant to ATM and CAZ-AVI (MIC > 256 mg/L) but susceptible to ATM + CAZ-AVI (CMI: 0.19 mg/L). The final diagnosis was a carbapenem-resistant *E. cloacae*-associated spacer super-infection. Definitive antibiotherapy was ATM (continuous infusion of 3 g in 150 ml of NaCl 0.09% per 12 h, twice a day), CAZ-AVI (continuous infusion of 3 g/0.75 g in 150 ml of NaCl 0.09% per 12 h, twice a day) as a continuous infusion using elastomeric infusor, colistin (4 MUI per 8 h), and daptomycin (850 mg per day).

After 2 weeks of colistin, the patient developed acute kidney injury with elevated eosinophils on blood test analyses. Immunoallergic interstitial nephritis associated with colistin was evocated: colistin was discontinued, and the patient received systemic corticosteroids. Renal function and eosinophil count improved shortly thereafter. ATM and CAZ plasmatic concentrations were monitored, and dosages were adapted accordingly to reach a steady-state concentration of at least four times the minimal inhibitor concentration (steady-state concentrations were 13.7 mg/ml and 85 mg/ml, respectively). No adverse event associated with ATM and CAZ was reported.

ATM, CAZ-AVI, and daptomycin were continued for 12 weeks. The patient underwent another surgery after 6 weeks of antibiotic treatment: the spacer was changed again (COPAL^®^ G+V). Further outcomes were favorable, and all antibiotics were discontinued at 12 weeks.

Unfortunately, upon the total knee arthroplasty reimplantation surgery performed after 3 months, pre-operative findings were evocative of persistent chronic infection, and microbiologic samples were positive for *Staphylococcus epidermidis*. Iterative surgery was required 1 month later because of unfavorable local outcomes and found evidence of *Candida parapsilosis* super-infection. There was no other occurrence of carbapenem-resistant *E. cloacae*-associated infection. Infection control was obtained after adequate antibiotic treatment, and a suspensive antimicrobial treatment with doxycycline and fluconazole was decided. Further outcomes were favorable at 24 months after the last surgery: no pain was reported, the patient could walk although there was a limitation in flexion angle, and there was no radiologic evidence of persistent chronic infection.

## Conclusion

The two patients described in this case series were both treated with ATM and CAZ-AVI for an MBL-producing *Enterobacterales*-associated BJI, with a favorable outcome despite the very high surgical and microbiological complexity of their cases.

Earlier cases of infections involving MBL-producing *Enterobacterales* treated with ATM and CAZ-AVI have already been reported (12) although most of them were bloodstream infections (including central line-associated bloodstream infections), UTIs, and LRTIs. In a multicentric prospective cohort in Italy and Greece (17) which compared ATM and CAZ-AVI to other active antibiotics in MBL-producing *Enterobacterales*-associated BSIs, the association of ATM and CAZ-AVI was associated with greater survival rates. Regarding BJI, few cases have been reported (13, 14), but favorable outcomes were reached with this association. Moreover, a case of VIM-producing *Pseudomonas aeruginosa* associated osteomyelitis successfully treated with ATM, CAZ-AVI, and amikacin was reported (18).

Although only two cases of BJIs treated favorably with ATM and CAZ-AVI are reported, our study provides further data supporting the use of this association in MBL-producing *Enterobacterales* involving BJIs. There are still challenges limiting the use of this association. First, ATM-AVI is not currently available without combining ATM and CAZ-AVI. Thus, treating patients with ATM and CAZ-AVI currently requires exposition to an unnecessary and potentially toxic CAZ treatment. Of note, the association ATM-AVI is currently under phase 3 investigation and might be commercially available in the future. Second, the administration of ATM and CAZ-AVI requires repeated intravenous infusions, which can be a hindrance to patient rehabilitation and hospital discharge. Nevertheless, our study provides data supporting the safety of at-home administration of ATM and CAZ-AVI with the use of elastomeric infusors.

In conclusion, 12 weeks treatment with the combination of ATM and CAZ-AVI through elastomeric infusors was associated with favorable outcomes in these two complex MBL-producing *Enterobacterales*-associated BJI patients. These results support further investigation of this association for routine treatment of such infections.

## Data availability statement

The original contributions presented in the study are included in the article/Supplementary material, further inquiries can be directed to the corresponding author.

## Ethics statement

The studies involving human participants were reviewed and approved by Hospices Civils de Lyon Ethics Committee. The patients/participants provided their written informed consent to participate in this study. Written informed consent was obtained from the individual(s) for the publication of any potentially identifiable images or data included in this article. Written informed consent was obtained from the participant/patient(s) for the publication of this case report.

## Author contributions

YM, AC, CK, and TF were involved in drafting the manuscript and revising it critically for important intellectual content. YM, AC, SB, AS, CH, SL, CB, and TF were involved in patient care and contributed to acquisition and interpretation of the data. FL, CK, and TR-G were responsible for microbiological analyses and contributed to acquisition and interpretation of the data. All authors read and approved the final manuscript.
