# Supplementary material for: Case report: Continuous infusions of ceftazidime-avibactam and aztreonam in combination through elastomeric infusors for 12 weeks for the treatment of bone and joint infections due to metallo-β-lactamase producing Enterobacterales
Source: Front Med (Lausanne). 2023 Aug 3;10:1224922. doi: 10.3389/fmed.2023.1224922 (PMC10435880; doi:10.3389/fmed.2023.1224922)
Supplement: Supplementary file 1 [file Data_Sheet_1.docx]

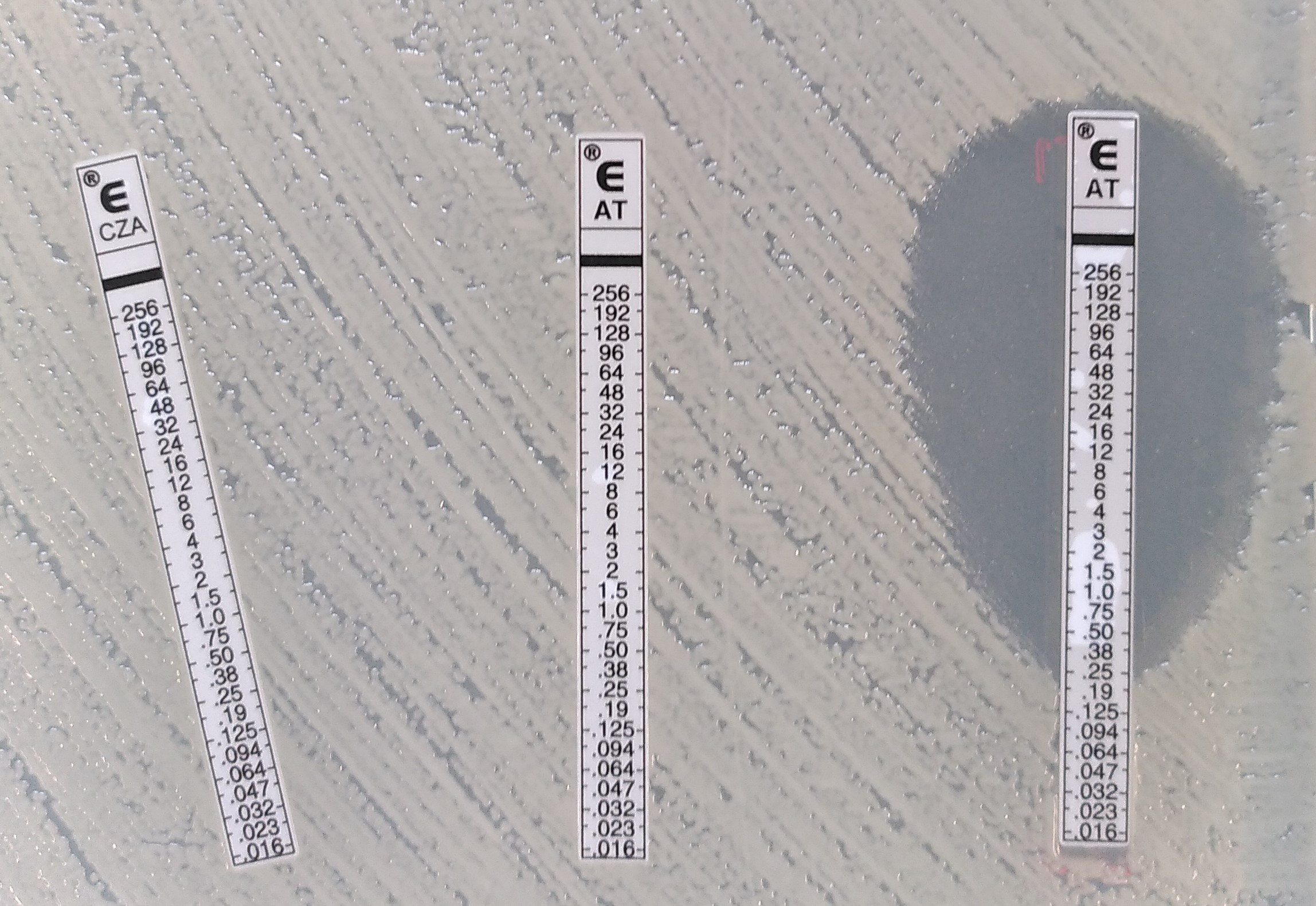


Supplementary figure 1 | MIC testing of a MBL-producing *Enterobacterales* strain. From left to right, CAZ-AVI strip alone, ATM strip alone and superposition of CAZ-AVI and ATM strips.
